# Cardiac Surgery Development in Cameroon: Unexpected Challenges From a Socio-Political Crisis

**DOI:** 10.3389/fcvm.2022.778075

**Published:** 2022-03-04

**Authors:** Charles Mve Mvondo, Alessandro Giamberti, Marcelin Ngowe Ngowe, Helen Anshoma Mbuoh, Italo Milocco, Hermann Nestor Tsague Kengni, Silvia Cirri, Alessandro Frigiola

**Affiliations:** ^1^Division of Cardiac Surgery, Shisong Cardiac Centre, St. Elizabeth Catholic General Hospital Shisong, Kumbo, Cameroon; ^2^Department of Surgery, Faculty of Medicine and Pharmaceutical Sciences, University of Douala, Douala, Cameroon; ^3^Division of Cardiac Surgery, Istituto di Ricovero e Cura a Carattere Scientifico San Donato Hospital, Milan, Italy; ^4^Division of Cardiology, Jordan Medical Services, Yaoundé, Cameroon; ^5^Department of Anesthesia and Intensive Care, Cardiothoracic Center, Istituto Clinico Sant'Ambrogio, Milan, Italy

**Keywords:** cardiac surgery, sub-Saharan Africa, political conflict, development, socio-political crisis

## Abstract

Despite the alarming and growing burden of cardiovascular diseases in sub-Saharan Africa (SSA), there is still a huge lack of specialised institutions in the region with a mean of one cardio-surgical unit for 33 million inhabitants. Despite the numerous efforts from humanitarian organisations made in recent years, the setting up of cardio-surgical units in the region remains challenging with regards to long-term sustainability. Indeed, besides the lack of financial resources, the insufficient local expertise in addition to the inadequate health infrastructure, unpredictable threats from external factors such as recurrent conflicts and humanitarian crises are still major concerns in an environment characterised by endemic socio-political instability. In Cameroon, located in the North West Anglophone region at 500 km from the capital, the cardiac centre of Shisong (CCS) is currently the lone cardio-surgical institution of the country. Fruit of a joint initiative of two Italian Non-governmental organisations namely, Bambini Cardiopatici nel Mondo (ABCnM) and Cuore Fratello (CF), and a local religious partner, the Tertiary Sisters of Saint Francis (TSSF), the CCS was faced with in the middle of a socio-political crisis that led to the urgent need of revision of the cardio-surgical project. The current paper reviews the impact of the ongoing socio-political crisis on the CCS over the past 3 years, in terms of clinical activities, staff perspectives, and long-term sustainability.

## Introduction

According to data from the literature, the highest prevalence of congenital and rheumatic heart diseases is reported in children living in sub-Saharan Africa (SSA) (1, 2). This carries an unbearable burden, as only a few specialised cardiac institutions are available in the region (3). Despite the great contributions from humanitarian organisations, the setting up of cardiology or cardiac surgery programs in the SSA has been minimal (4–7). Indeed, besides the challenges related to the scarcity of financial resources, the lack of infrastructures, and local expertise, long-term sustainability is impacted by the unpredictable socio-political dynamics of the local environment (8–10). Thus, a proper understanding of the regional and socio-political patterns is critical when seeking sustainable projects in the region.

In Cameroon, the lone cardio-surgical project of the country faced unexpected challenges following a socio-political crisis requiring prompt measures to preserve the institution's objectives.

## Background

### Cameroon

Cameroon is often referred to as “Africa in miniature” mainly for her cultural richness and geographical diversity. The country's two official languages are English and French. It is the largest country in the central Africa sub-region with ~25 million inhabitants and is geographically and administratively divided into 10 regions. Whereas, two (northwest and southwest regions) are of Anglophone influence, eight are of francophone culture. Over the past years, the country has been under security tensions due to the incursions of the Boko Haram terrorist movement at the boundaries with Nigeria and sporadic tensions from Central African rebels. Since 2016, growing social protests in the two Anglophone (The Anglophone Crisis) regions have gradually involved in an armed conflict opposing governmental forces and separatist armed groups.

The estimated Gross National Income Per capita of the country in 2019 was estimated at 3,730 USD with 30% of the population living below the threshold of poverty.

Data from the WHO fact sheets have reported a ratio of 1.1 physicians for 10,000 population in the country^1^. There is no free healthcare coverage system and health services are directly afforded by patients and a small number are covered by private insurance.

Cameroon is probably the country with a major number of cardiologists among the central African countries. Approximately 80 cardiologists associated with the “Cameroonian Cardiology Society” were registered in the country in 2018, the majority assigned to the major hospitals in the two main cities Yaoundé and Douala. Interventional cardiology and cardiac surgery specialities were only reported at the cardiac centre of Shisong (CCS), the lone cardio-surgical unit performing regular surgeries over the last decade (4).

### The Cardiac Centre of Shisong

**Figure 1:**
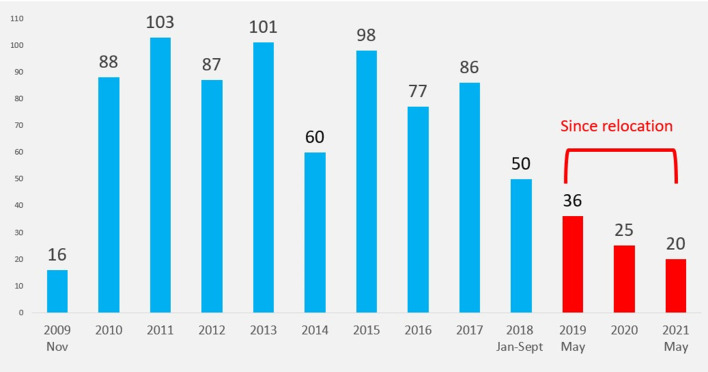
Surgical activities at the Shisong Cardiac Centre since 2019 (before and after relocation). The y-axis represents the number of cardiac surgeries.

The CCS is a cardio-surgical department of the St. Elizabeth Catholic General Hospital of Shisong (SECGH) which is located in the city of Kumbo in the northwest zone, one of the two Anglophone regions in Cameroon. The project is the fruit of a collaboration between two Italian non-governmental organisations, namely, Bambini Cardiopatici Nel Mondo (ABCnM) and Cuore Fratello (CF), and a local Cameroonian partner, the Tertiary Sisters of St. Francis (TSSF), a Franciscan Catholic religious order with more than 70 years of experience in providing healthcare services in the country. The CCS was inaugurated in November 2009 as the lone cardio-surgical centre in the whole Central African Economic and Monetary Community (CEMAC), an area with an estimated 40 million people (4, 11). The project's main objective was to build local autonomy through an extended and continue a training program for the locals, mainly in the fields of cardiac surgery, interventional cardiology, and electrophysiology. From the inauguration, the clinical activities are carried out by both visiting teams and the resident staff. A total of 847 open-heart surgeries including adult and paediatric cases have been performed at the institution since its inauguration (Figure 1).

### The Socio-Political Crisis

Cameroon carries a 60-year legacy of colonisation that was built under the British and French occupation. The long-term unsolved socio-cultural and political divergences between the French majority (80%) and the English minority (20%) have partly jeopardised the hope for effective and immediate post-independence unity. In November 2016, protests from lawyers and teachers in the Anglophone regions (northwest and southwest) demanding the restoration of educational and judiciary systems specificities have revealed the latent frustration based on linguistic, ethnic, and sociocultural differences with the French counterpart. The failure to promptly address the protests has led to violent escalation fuelled by an opportunistic separatist campaign. Over the past 3 years, the armed conflict opposing governmental forces and separatist armed groups have caused more than 60,000 refugees in neighbouring east Nigeria, 600,000 internally displaced people, and ~4,000 deaths. A countless number of villages, social infrastructures especially schools, markets, and medical institutions have been burned, with progressive destruction of the local economy^2^ (12). The CCS located in the northwest region was at the epicentre of the violence undergoing significant changes in the course of its activities.

## The Impact of the Conflict on the Cardiac Centre

A total of 847 open-heart surgeries have been performed at CCS since its inauguration in 2009. The number of cases performed every year is reported in Figure 1.

Between September 2018 and May 2019, no surgical activities were performed in our institution following the drastic drop in patient attendance.

**Figure 2:**
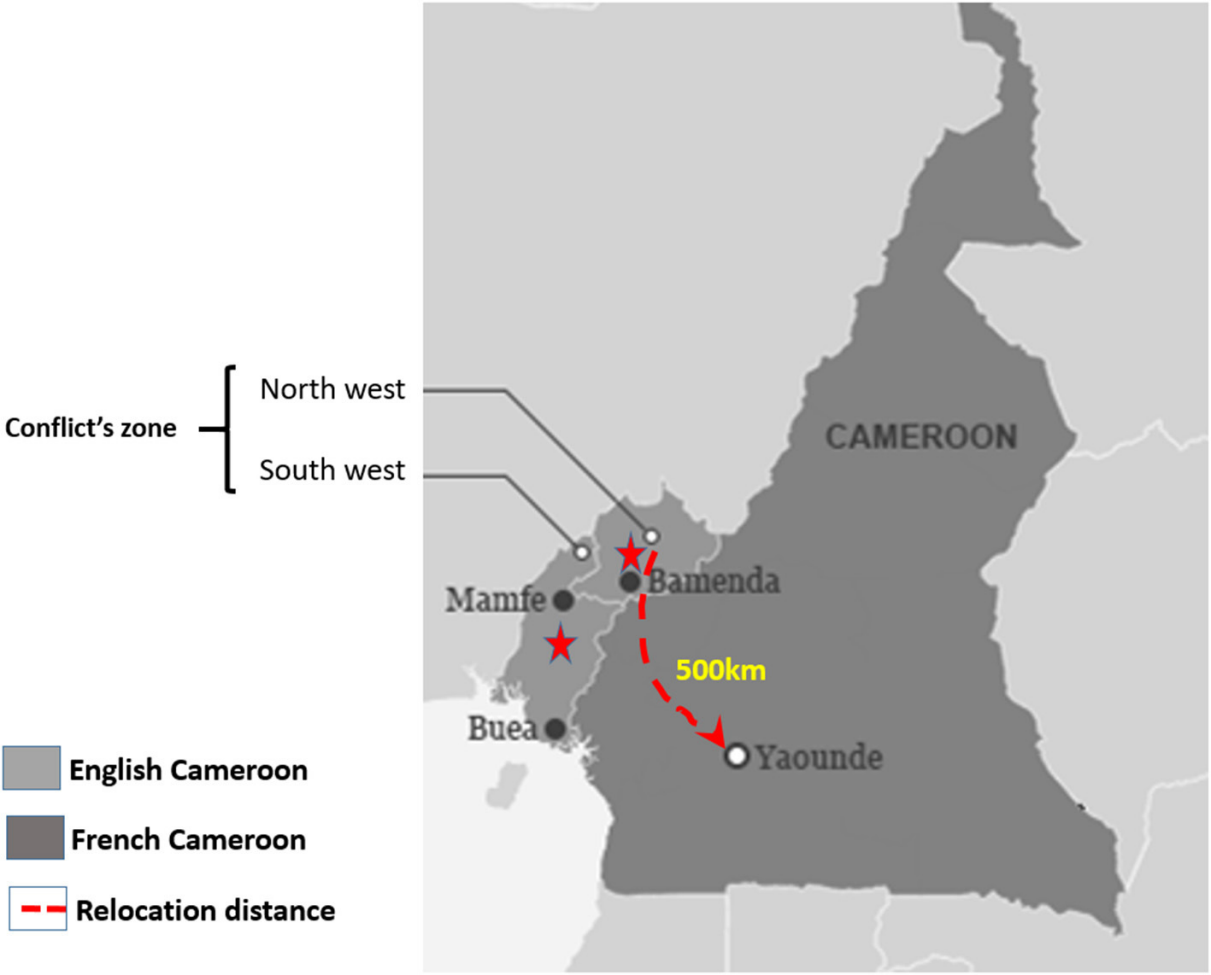
Geographic illustration of the location of the cardiac centre of Shisong (CCS) and the successive relocation site.

Since May 2019, the board decision was made to relocate the cardio-surgical unit of the CCS; this included a team of 21 members from administrative, technical, and medical staff that was relocated (Outreach-CCS, OCCS), 500 km away in the country capital, Yaoundé (Figure 2). The move was conducted through a private-private partnership between the CCS and a private clinic (The Jordan Medical Services, JMS). A technical renovation of the OCCS existing operating theatres was necessary for addition to an extension of the intensive care unit building to fit the standards for a cardiac surgery unit. The new unit was composed of an operating theater and a 5-bed Intensive Care Unit. The movement of a catheterisation laboratory was not achieved due to technical difficulties and the prohibitive costs during the dismantling, transportation, and installing process of the CCS angiograph.

The whole relocation process was completed 3 months after the board decision was taken and additional 2 weeks were needed to resume the surgical activities in the new site.

From the relocation of the cardio-surgical unit to date (May 2019–2021), a total of 81 patients have been operated upon at the OCCS. The indication for surgery was mainly acquired valvulopathies and less complex congenital heart defects. In the absence of a cathlab, a preoperative coronary angio-CT scan was performed in patients with no risk factors for coronary disease undergoing elective acquired heart disease surgery. When compared to the 3 years before the armed conflict (2015–2017), there has been a significant decrease of surgical activities by 62% (2016–2018: 213 cases; 2019–2021: 81 surgeries).

**Figure 3:**
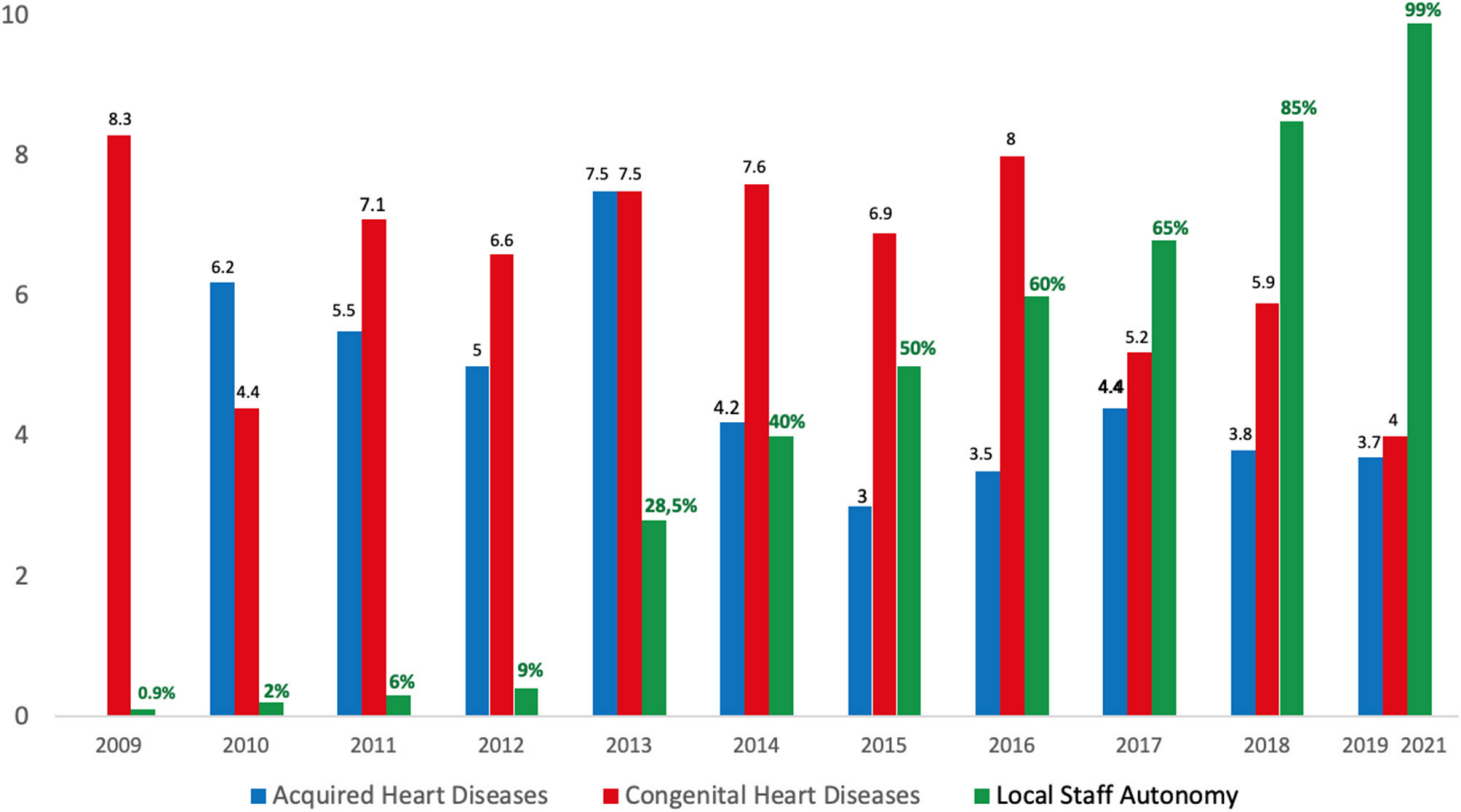
The bar graph shows the hospital mortality (%) rate after surgery for acquired heart disease (blue bar) and congenital heart disease (red bar). The green bar shows the rate of local staff surgical autonomy.

From 2018 to 2021 all the surgeries were performed by the resident local team. There was a growth in local team autonomy from 65% in 2018 to 100% during the relocation period (Figure 3).

## Reflection

Despite the SSA having the world's highest burden of cardiovascular diseases in the paediatric and young population (1, 2), the access to specialised cardiovascular care remains inadequate with a mean of one cardio-surgical centre for 33 million people living in the region (3, 13). In recent years, a great number of cardio-surgical programs in the SSA have been developed with the major contribution of humanitarian organisations (3). However, some criticisms have been raised on the development of cardio-surgical programs through humanitarian projects, for their controversial results in terms of effectiveness and long-term sustainability, which have questioned their real epidemiologic impact. In series by Yankah et al. and Zilla et al. (3, 13), a poor surgical volume between 2 and 4 surgeries per million was reported despite a decade of activities, with suboptimal results in building local staff autonomy. While cardio-surgical projects are often challenged by the poor resources allocation and the lack of local expertise (4, 5), the pernicious impact of the surrounding socio-cultural and political environment has been rarely addressed. Thus, along with the rampant corruption and the lack of political willingness, the unpredictable progression of social tensions are severe threats to the sustainability of projects (14–16). Indeed, more than six decades of recurrent conflicts in the SSA have caused various humanitarian crises (17) with disastrous consequences on local healthcare systems. This has included the overcrowding and destruction of medical facilities, the disruption of drugs supply, and the massive exodus of health workers due to safety concerns (18). Data from WHO and others (19, 20) have reported the destruction of the existing healthcare facilities to up to 65% during conflicts in several SSA countries. Therefore, an accurate feasibility analysis must provide predictive indicators of social threats to facilitate the implementation of every health care project. When we started the project in 2001, the choice to create the CC in Shisong was made because we felt that being away from large cities with a huge administrative burden and conflicts of interest was a greater guarantee of success.

In our case, three potential indicators could have raised major interest in predicting the social conflict. First, the main project's infrastructure was built in a politically sensitive area. Latent tensions from the reported marginalisation of the 20% Anglophone minority have progressively grown over the past five decades following the colonial era (21, 22). Although the region was peaceful throughout the project development, the choice of the site was debatable. Secondly, the poor accessibility and long distance from the main cities (>500 km, >10–12 h road trip) to the Hospital was a weak point in terms of logistics facilities (hospital supply, patient's referral, and safe transportation of foreign volunteers) and security concerns as seen during the armed conflict. Indeed, the cessation of hospital activities due to both the drop in patient admission, the disruption of material supply, and the cessation of volunteer's missions were all linked to the recurrent road blockages by armed groups. Lastly, the absence of a practical private-public network between the project and the main healthcare and academic institutions was a limiting factor. Indeed, an effective private-public collaboration could have been supportive during the conflict by facilitating the relocation process.

With the violent escalation of the conflict and the suspension of speciality units' activities, an urgent relocation out of the conflict zone was more than a necessity. The hospital accessibility to patients from others regions had become hazardous. The poor hospital attendance and technical issues arising from the recurrent shortage of electricity and oil supply have fuelled the uncertainty for an early return to normality. Additionally, an increasing financial burden led to the gradual release and exodus of the staff. Thus, the prompt restoration of specialised activities out of the conflict area became critical despite some reluctance related to the context. The decision to “transfer” the lone cardio-surgical unit of the country from the Anglophone conflict zone to the French capital at the peak of the crisis was symbolically sensitive and far from being unanimous even among the project stakeholders. Other criticisms were the risks related to the disassembly and reinstallation process of the complex equipment considering the potential damage or hijacking during the transportation phase along the conflict road.

The relocation strategy has allowed us to restore a functional surgical unit despite the reduction of hospital activities by more than 62% at the peak of the crisis. Moreover, the surgical procedures were fully carried out by the local staff as no foreign teams' campaigns could take place due to security issues and the progression of the COVID-19 pandemic over the last 2 years. The full functioning of the resident team emphasises the importance of supporting local autonomy to build long-term sustainability. From representing <60% of the surgical activities in 2016, the local team currently provides 100% of the surgeries of the institution covering the entire spectrum of acquired heart diseases and less complex congenital heart diseases in children > 5 kg (Atrial and ventricular septal defects, Patent Ductus Arteriosus, partial atrioventricular canal defects, less complex Tetralogy of Fallot, etc.). To date, more complex congenital lesions such as complete atrioventricular canal defects in patients with Down's syndrome, transposition of the great arteries are still referred abroad. On the other hand, as the new unit was located in a more central region with a high density of private and public hospitals and transportation facilities, the collaboration with surrounding specialists has greatly improved with regards to patient referral.

In summary, the endemic socio-political instability in the sub-Saharan region remains an insidious threat to local development, including for highly specialised healthcare projects such as cardiac surgery. Feasibility studies prior to the implementation of each project should therefore provide accurate data on local socio-political and cultural dynamics. Strategies aiming at developing local staff autonomy and effective collaboration with local government and major healthcare institutions in the region are key factors to support long-term sustainability.

## Data Availability Statement

The raw data supporting the conclusions of this article will be made available by the authors, without undue reservation.

## Author Contributions

All authors contributed to conception and design of the study, read, and approved the submitted version.

## Funding

This work was partially supported by the NGO Bambini Cardiopatici nel Mondo.

## Conflict of Interest

The authors declare that the research was conducted in the absence of any commercial or financial relationships that could be construed as a potential conflict of interest.

## Publisher's Note

All claims expressed in this article are solely those of the authors and do not necessarily represent those of their affiliated organizations, or those of the publisher, the editors and the reviewers. Any product that may be evaluated in this article, or claim that may be made by its manufacturer, is not guaranteed or endorsed by the publisher.

## Footnotes

^1^https://www.who.int/workforcealliance/countries/cmr/en/ (accessed December 26, 2021).

^2^The_anglophone_problem_an_analysis_of_the_anglophone_crisis_in_Cameroon https://www.researchgate.net/publication/337324438 (accessed December 26, 2021).
